# Metabolomics revealed diurnal heat stress and zinc supplementation‐induced changes in amino acid, lipid, and microbial metabolism

**DOI:** 10.14814/phy2.12676

**Published:** 2016-01-12

**Authors:** Lei Wang, Pedro E. Urriola, Zhao‐hui Luo, Zachary J. Rambo, Mark E. Wilson, Jerry L. Torrison, Gerald C. Shurson, Chi Chen

**Affiliations:** ^1^ Department of Food Science and NutritionUniversity of MinnesotaSaint PaulMinnesota; ^2^ Department of Animal ScienceUniversity of MinnesotaSaint PaulMinnesota; ^3^ Zinpro CorporationEden PrairieMinnesota

**Keywords:** Heat stress, metabolism, metabolomics, zinc supplementation

## Abstract

Heat stress (HS) dramatically disrupts the events in energy and nutrient metabolism, many of which requires zinc (Zn) as a cofactor. In this study, metabolic effects of HS and Zn supplementation were evaluated by examining growth performance, blood chemistry, and metabolomes of crossbred gilts fed with ZnNeg (no Zn supplementation), ZnIO (120 ppm ZnSO_4_), or ZnAA (60 ppm ZnSO_4_ + 60 ppm zinc amino acid complex) diets under diurnal HS or thermal‐neutral (TN) condition. The results showed that growth performance was reduced by HS but not by Zn supplementation. Among measured serum biochemicals, HS was found to increase creatinine but decrease blood urea nitrogen (BUN) level. Metabolomic analysis indicated that HS greatly affected diverse metabolites associated with amino acid, lipid, and microbial metabolism, including urea cycle metabolites, essential amino acids, phospholipids, medium‐chain dicarboxylic acids, fatty acid amides, and secondary bile acids. More importantly, many changes in these metabolite markers were correlated with both acute and adaptive responses to HS. Relative to HS‐induced metabolic effects, Zn supplementation‐associated effects were much more limited. A prominent observation was that ZnIO diet, potentially through its influences on microbial metabolism, yielded different responses to HS compared with two other diets, which included higher levels of short‐chain fatty acids (SCFAs) in cecal fluid and higher levels of lysine in the liver and feces. Overall, comprehensive metabolomic analysis identified novel metabolite markers associated with HS and Zn supplementation, which could guide further investigation on the mechanisms of these metabolic effects.

## Introduction

High ambient temperature‐elicited heat stress (HS) is a detrimental pathophysiological event. Besides its threat to human health (Changnon et al. 1996; Leon and Helwig 2010), HS also negatively affects the growth performance, reproduction, and health status of farm animals (Hansen 2009; Renaudeau et al. 2011). Annual cost of HS on the US swine industry is over $300 million (St‐Pierre et al. 2003). Under high ambient temperature, inadequate release of body heat elevates core body temperature, leading to extensive systemic changes and physiological responses. In cardiovascular and respiratory systems, HS elevates cardiac and respiratory rates and causes cutaneous vasodilation (Kellogg et al. 1998; Patience et al. 2005). Systemic inflammatory responses could also be triggered by HS‐induced translocation of bacteria and endotoxins across compromised tight junctions in the gut (Dokladny et al. 2006; Pearce et al. 2014). The metabolic system is also greatly affected by HS‐induced alterations in metabolism‐related hormones, genes, proteins, and metabolites (Aggarwal and Upadhyay 2013; Baumgard and Rhoads 2013). The well‐known effects of HS on metabolism‐related hormones, genes, and proteins include attenuated releases of thyroid and growth hormones, which decrease basal metabolic rate (McGuire et al. 1991; Aggarwal and Upadhyay 2013), and alter expression of genes and proteins involving in energy and nutrient metabolism (Rhoads et al. 2011; Stallings et al. 2014; Victoria Sanz Fernandez et al. 2015). The effects of HS on metabolites have also been examined by both metabolomics (Malmendal et al. 2006; Ippolito et al. 2014) and targeted analysis of specific metabolites in AA, lipid, and carbohydrate metabolic pathways (Baumgard and Rhoads 2013; Belhadj Slimen et al. 2015). However, the short‐term exposure in these metabolomics studies aimed to mimic acute exposure in humans but not extended exposure in animals, while targeted metabolite analysis lacks the capacity to cover the metabolome and to identify novel metabolic changes. Therefore, the metabolic responses to reoccurring diurnal HS remains largely unexplored.

Zinc (Zn), as an essential mineral, is commonly supplemented in animal feeds to maintain health status and achieve optimal growth performance. Inorganic Zn, mainly zinc oxide (ZnO) and zinc sulfate (ZnSO_4_), is widely used for this purpose, though the use of organic zinc has also been explored in recent years after observing synergistic effects of AAs in Zn absorption (Wapnir and Stiel 1986; Hollis et al. 2005; Sahin et al. 2005). In practice, the pharmacological levels of Zn have been widely used to improve the growth performance of young pigs (Carlson et al. 1999). Zn has also been explored as a supplement for protecting animals against the adverse effects of HS (Sahin and Kucuk 2003; Sahin et al. 2009). The protective effects of Zn have been partially attributed to its antioxidant property (Powell 2000), while its growth promotion effects are likely due to its functions as the cofactor of insulin, transcriptional factors, and metabolic enzymes (Lynch et al. 2001; Hedemann et al. 2006; Maret 2013). Despite these established functions of Zn in metabolism, whether Zn supplementation could affect HS‐induced metabolic changes is largely unknown.

Through growth performance, blood chemistry, and liquid chromatography‐mass spectrometry (LC‐MS)‐based metabolomic analysis of serum, hepatic extract, cecal fluid, fecal extract, and urine samples, the present study aims to characterize metabolic effects of diurnal HS challenge as well as to determine whether Zn supplementation could affect metabolic responses to HS. Comprehensive examination of the metabolomes in these biological samples led to identification of time‐ and site‐specific metabolite markers that are associated with HS and Zn‐induced changes in AA, lipid, and microbial metabolism.

## Materials and Methods

### Chemicals

The following chemicals were used: LC‐MS‐grade water and acetonitrile (ACN) (Fisher Scientific, Houston, TX); 2‐hydrazinoquinoline (HQ) and triphenylphosphine (TPP) (Alfa Aesar, Ward Hill, MA); 2,2′‐dipyridyl disulfide (DPDS) (MP Biomedicals, Santa Ana, CA); and dansyl chloride (DC) and n‐butanol (Sigma‐Aldrich, St. Louis, MO). The metabolite standards used for structural confirmation were from Sigma‐Aldrich, Fisher Scientific, Alfa Aesar, Ark Pharm (Libertyville, IL), Frontier Scientific (Logan, UT), and Steraloids (Newport, RI), respectively.

### Animals and feeds

Forty‐eight crossbred gilts (prebreeding female pigs) in growing phase, with initial body weight (BW) of 71 ± 9 kg, were assigned to 1 of 6 treatments in a 2 temperatures × 3 feeds factorial design (8 pigs/treatment) at the Iowa State University Swine Nutrition Farm (Ames, IA). All three feeds (ZnNeg: no Zn supplementation; ZnIO: 120 ppm ZnSO_4_; and ZnAA: 60 ppm ZnSO_4_ + 60 ppm Availa^®^Zn zinc AA complex) were prepared by mixing three mineral premixes with corn–soybean meal, respectively. The nutrient contents in formulated diets met or exceeded the National Research Council (NRC) requirements (2012) for energy, essential AAs, minerals, and vitamins (Table S1). The concentrations of minerals in the premixes and diets were measured. The Zn levels in ZnIO and ZnAA were 154 and 143 ppm, respectively. The presence of low‐level Zn in ZnNeg diet suggested that corn and soybean meal provided about 30 ppm Zn in all three experimental diets (Table S2). The animal study protocol was reviewed and approved by the Iowa State University Institutional Animal Care and Use Committee (IACUC). The mineral contents of premixes and feeds were measured by the Minnesota Valley Testing Lab (New Ulm, MN).

### Animal experiment

**Figure 1:**
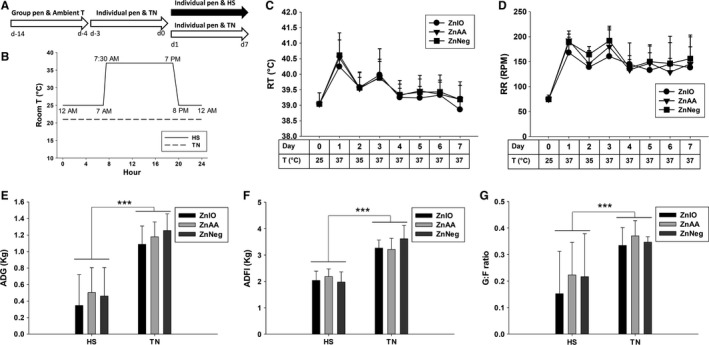
Effects of HS and Zn supplementation on physiological responses and growth performance of growing pigs. (A) Timeline of 21‐day feeding experiment. Pigs had ad libitum access to water and diet and were fed with one of assigned diets (ZnNeg, ZnIO, or ZnAA) through entire feeding experiment. (B) Designed daily temperature settings in TN and HS treatments. (C–D) Average RT and RR of pigs after 2 h of HS. *T* is the room temperature when RT and RR were measured. (E–G) ADG, ADFI, and G:F ratio of pigs under HS and TN conditions. Significant differences between TN and HS are labeled as ***P <* *0.001.

The feeding experiment was divided into three periods (Fig. 1A). From day −14 to −4, pigs were housed in group pens and fed with one of assigned diets. From day −3 to 0, pigs were moved to two separate environment rooms and acclimated to the individual pens for 4 days under thermal‐neutral (TN) condition (21°C room temperature). From day 1 to 7, diurnal HS (12 h 25°C + 12 h 37°C) was applied in one environment room while TN was maintained in the other room (Fig. 1B). During whole experiment, pigs were ad libitum access to water and diet. For pigs under HS treatment, rectal temperature (RT) and respiratory rate (RR) were monitored five times daily at 9 am, 11 am, 1 pm, 3 pm, and 5 pm for the first 3 days. RT was measured using a digital thermometer; 41°C is the threshold RT for mitigation treatment following IACUC protocol. RR was determined by counting flank movements for 1 min. BW was measured on day −14, −4, and 0 and then daily during day 1–7. Feed intake was measured between day −4 and 0 and then daily from day 1 to 7.

### Sample collection

Blood samples were collected via jugular venipuncture using Vacutainer tubes (Becton Dickinson, Franklin Lakes, NJ) at 1 PM of the sampling days (day 0, 2, and 7 of TN treatment; day 0, 1, 2, 5, and 7 of HS treatment). After the collection, the samples were allowed to clot at room temperature for 20 min and then placed on ice. Serum were obtained by centrifugation of blood samples at 1400 × *g* for 10 min and stored at −80°C. On day 7, liver, cecal fluid, and fecal samples were collected immediately after killing and snap‐frozen in liquid nitrogen. Urine samples were drawn from bladder using syringes. All these samples were stored at −80°C until further analysis.

### Blood chemistry

The levels of cholesterol, triglycerides, glucose, creatinine, blood urea nitrogen (BUN), and iron in serum samples were measured using the reagent kits (Pointe Scientific, Canton, MI). Serum Zn level was determined using a reagent kit (Sigma‐Aldrich).

### Metabolomics

LC‐MS‐based metabolomic analysis comprises sample preparation, chemical derivatization, LC‐MS analysis, data deconvolution and processing, multivariate data analysis (MDA), and marker characterization and quantification (Chen et al. 2007).

### Sample preparation

For serum samples, deproteinization was conducted by mixing one volume of serum with 19 volumes of 66% aqueous ACN and then centrifuging at 18,000 × *g* for 10 min to obtain the supernatants. Liver tissue samples were fractionated using a modified Bligh and Dyer method (Bligh and Dyer 1959). Briefly, 100 mg of liver sample was homogenized in 0.5 mL of methanol and then mixed with 0.5 mL of chloroform and 0.4 mL of water. After 10‐min centrifugation at 18,000 × *g*, upper aqueous fraction was harvested while chloroform fraction was dried by nitrogen and then reconstituted in *n*‐butanol. After precipitating insoluble content by centrifugation, cecal fluid samples were further diluted with 4 volumes of 50% aqueous ACN and then centrifuged at 18,000 × *g* for 10 min to obtain the supernatants. Feces samples were mixed with 50% aqueous ACN in 1:9 (w/v) ratio and then centrifuged at 18,000 × *g* for 10 min to obtain fecal extract supernatants. Urine samples were processed by mixing one volume of urine with 4 volumes of 50% aqueous ACN and then centrifuged at 18,000 × *g* for 10 min to obtain the supernatants.

### Chemical derivatization

For detecting the metabolites containing amino functional group in their structures, the samples were derivatized with DC prior to the LC‐MS analysis. Briefly, 5 *μ*L of sample or standard was mixed with 5 *μ*L of 100 μmol/L *p*‐chlorophenylalanine (internal standard), 50 *μ*L of 10 mmol/L sodium carbonate, and 100 *μ*L of DC solution (3 mg/mL in acetone). The mixture was incubated at 25°C for 15 min and centrifuged at 18,000 × *g* for 10 min, and the supernatant was transferred into a HPLC vial for LC‐MS analysis. For detecting carboxylic acids, aldehydes and ketones, the samples were derivatized with HQ prior to the LC‐MS analysis (Lu et al. 2013). Briefly, 2 *μ*L of sample was added into a 100 *μ*L of freshly prepared ACN solution containing 1 mmol/L DPDS, 1 mmol/L TPP, and 1 mmol/L HQ. The reaction mixture was incubated at 60°C for 30 min, chilled on ice, and then mixed with 100 *μ*L of ice‐cold H_2_O. After centrifugation at 18,000 × *g* for 10 min, the supernatant was transferred into a HPLC vial for LC‐MS analysis.

### LC‐MS analysis

A 5 *μ*L of aliquot prepared from serum, hepatic extract, cecal fluid, fecal extract, or urine sample was injected into an Acquity ultra‐performance liquid chromatography (UPLC) system (Waters, Milford, MA) and separated in a BEH C18 column (Waters). The mobile phase for underivatized and DC‐derivatized samples used a gradient ranging from water to 95% aqueous ACN containing 0.1% formic acid over a 10 min run, while the mobile phase for HQ‐derivatized samples contained A: H_2_O containing 0.05% acetic acid (v/v) and 2 mmol/L ammonium acetate; B: H_2_O:ACN = 5:95 (v/v) containing 0.05% acetic acid (v/v) and 2 mmol/L ammonium acetate. LC eluant was introduced into a Xevo‐G2‐S quadrupole time‐of‐flight mass spectrometer (QTOFMS, Waters) for accurate mass measurement and ion counting. Capillary voltage and cone voltage for electrospray ionization were maintained at 3 kV and 30 V for positive‐mode detection or at −3 kV and −35 V for negative‐mode detection, respectively. Source temperature and desolvation temperature were set at 120°C and 350°C, respectively. Nitrogen was used as both cone gas (50 L/h) and desolvation gas (600 L/h) and argon as collision gas. For accurate mass measurement, the mass spectrometer was calibrated with sodium formate solution with mass‐to‐charge ratio (*m/z*) of 50–1000 and monitored by the intermittent injection of the lock mass leucine enkephalin ([M + H]^+^ = *m/z* 556.2771 and ([M − H]^−^ = *m/z* 554.2615) in real time. Mass chromatograms and mass spectral data were acquired and processed by MassLynx^TM^ software (Waters) in centroided format. Additional structural information was obtained tandem MS (MSMS) fragmentation with collision energies ranging from 15 to 40 eV.

### Data deconvolution and processing

After data acquisition in the UPLC‐QTOFMS system, chromatographic and spectral data of samples were deconvoluted by MarkerLynx^TM^ software (Waters). A multivariate data matrix containing information on sample identity, ion identity (retention time and *m/z*), and ion abundance was generated through centroiding, deisotoping, filtering, peak recognition, and integration. The intensity of each ion was calculated by normalizing the single ion counts (SIC) versus the total ion counts (TIC) in the whole chromatogram.

### MDA

The processed data matrix was exported into SIMCA‐P+^TM^ software (Umetrics, Kinnelon, NJ), transformed by *Pareto* scaling, and then analyzed by unsupervised principal components analysis (PCA) and supervised partial least squares‐discriminant analysis (OPLS‐DA). Major latent variables in the data matrix were determined as the principal components of PCA model, and the relationships among examined samples were described in the scores scatter plot. Metabolite markers of HS exposure and Zn supplementation were identified by analyzing ions contributing to sample separation in OPLS‐DA model. After *Z* score transformation, the concentrations or relative abundances of identified metabolite markers in examined samples were presented in the heat maps generated by the R program (http://www.R-project.org), and the correlations among these metabolite markers were defined by hierarchical clustering analysis (HCA).

### Marker characterization and quantification

The chemical identities of metabolite markers were determined by accurate mass measurement, elemental composition analysis, searching Human Metabolome Database (HMDB), Kyoto Encyclopedia of Genes and Genomes (KEGG), and Lipid Maps databases using MassTRIX search engine (http://masstrix3.helmholtz-muenchen.de/masstrix3/) (Suhre and Schmitt‐Kopplin 2008), MSMS fragmentation, and comparisons with authentic standards if available. Individual metabolite concentrations were determined by calculating the ratio between the peak area of metabolite and the peak area of internal standard and fitting with a standard curve using QuanLynx^TM^ software (Waters).

### Statistical analyses

The statistical significances among diet and treatment groups were analyzed using the PROC MIXED procedure of SAS version 9.1 (SAS Institute, Cary, NC). Data are reported as least squares means (LSMEANS). Student's *t*‐test was used for pairwise comparisons of two time points within a treatment. The PROC GLM procedure of SAS was used to compare three diet groups. Differences are considered significant if *P *<* *0.05 and as a tendency if 0.05 ≥ *P *≤* *0.10.

## Results

### Physiological responses and growth performance

Under TN condition, average RT of pigs was 39.0 ± 0.4°C, and their average RR was 75 ± 8 breaths per minute (BPM). After diurnal HS challenge, average RT and RR were greatly increased (Fig. 1C–D). On day 1 of HS, average RT rose up to 40.5 ± 0.6°C after 2 h of 37°C HS, and average RR became 183 ± 25 BPM (Fig. 1C–D). Furthermore, after 4 h of 37°C exposure on day 1, half of all 24 pigs under HS challenge had their RT higher than 41°C, which is the threshold temperature for the mitigation treatment required by approved IACUC protocol. This situation led to the adjustment of ambient temperature to 35–36°C on day 1–3 to avoid heat stroke. However, on day 4–7, RT of HS group became comparable to that of TN group, and hence, ambient temperature was maintained at 37°C during daily 12‐h HS challenge (Fig. 1C). In contrast, RR of HS group was still significantly higher than its value in TN group (Fig. 1D). Overall, these observations suggested that HS led to an initial acute response followed by a gradual physiological acclimation.

HS reduced average daily gain (ADG), average daily feed intake (ADFI), and gain:feed ratio (G:F ratio), while Zn supplementation did not significantly alter live performance metrics (Fig. 1E–G).

### Blood chemistry

**Figure 2:**
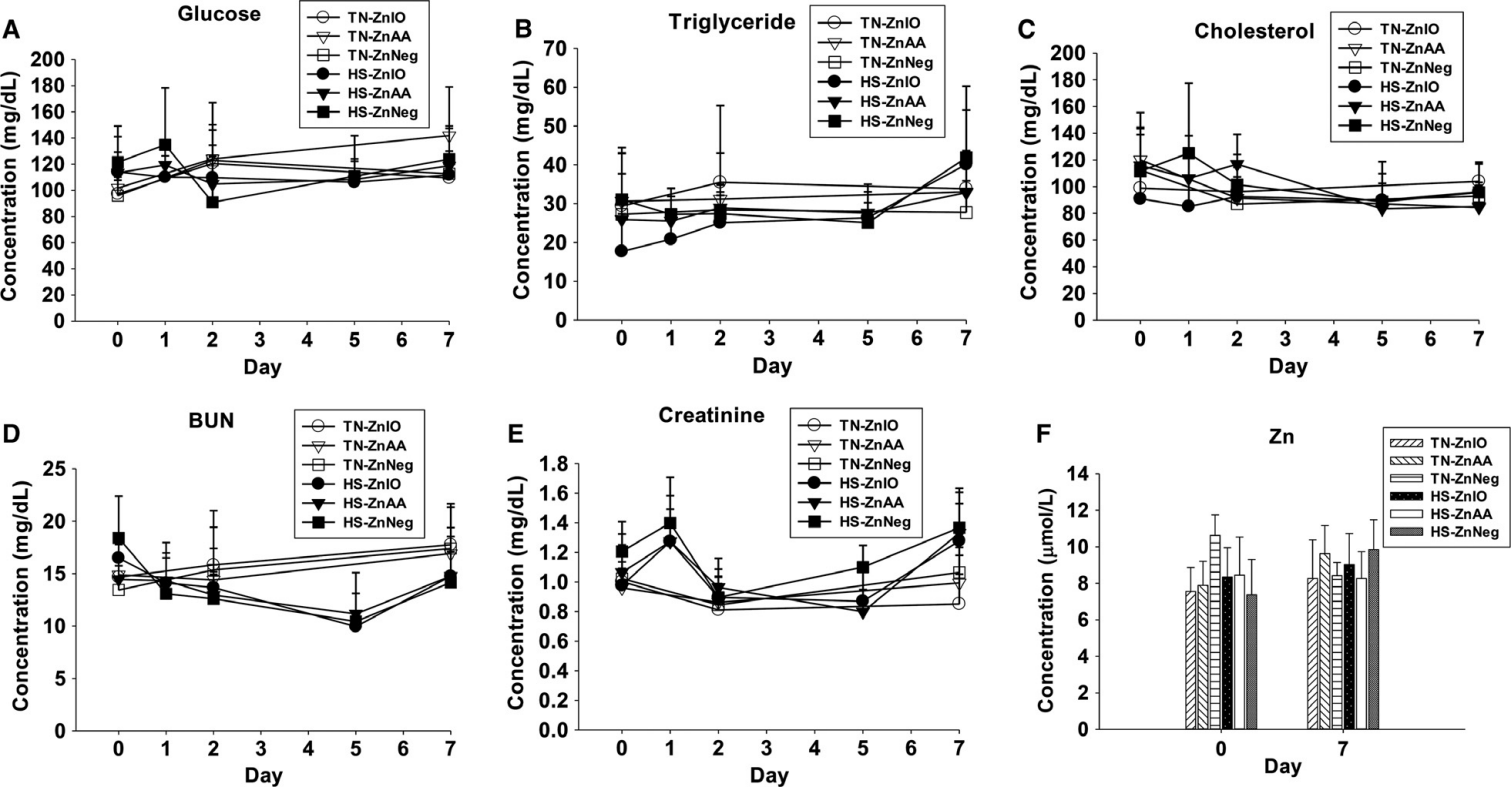
Effects of HS and Zn supplementation on blood chemistry of growing pigs. (A) Serum glucose, (B) Serum triglycerides, (C) Serum cholesterol, (D) Serum BUN, (E) Serum creatinine, (F) Serum Zn.

Despite some day‐to‐day fluctuation, serum glucose, triglycerides, and cholesterol levels were not significantly affected by either HS or Zn supplementation during 1‐week treatment (Fig. 2A–C). In contrast, serum BUN level was gradually decreased by HS from day 1 to 5, while serum creatinine level was significantly higher on days 1 and 7 of HS than its level on day 0 (Fig. 2D–E). Furthermore, serum Zn level on days 0 and 7 was comparable among all treatment groups (Fig. 2F). Serum iron level was also not affected by Zn supplementation and HS during 1‐week treatment (data not shown).

### Serum metabolome during 1‐week HS challenge

**Figure 3:**
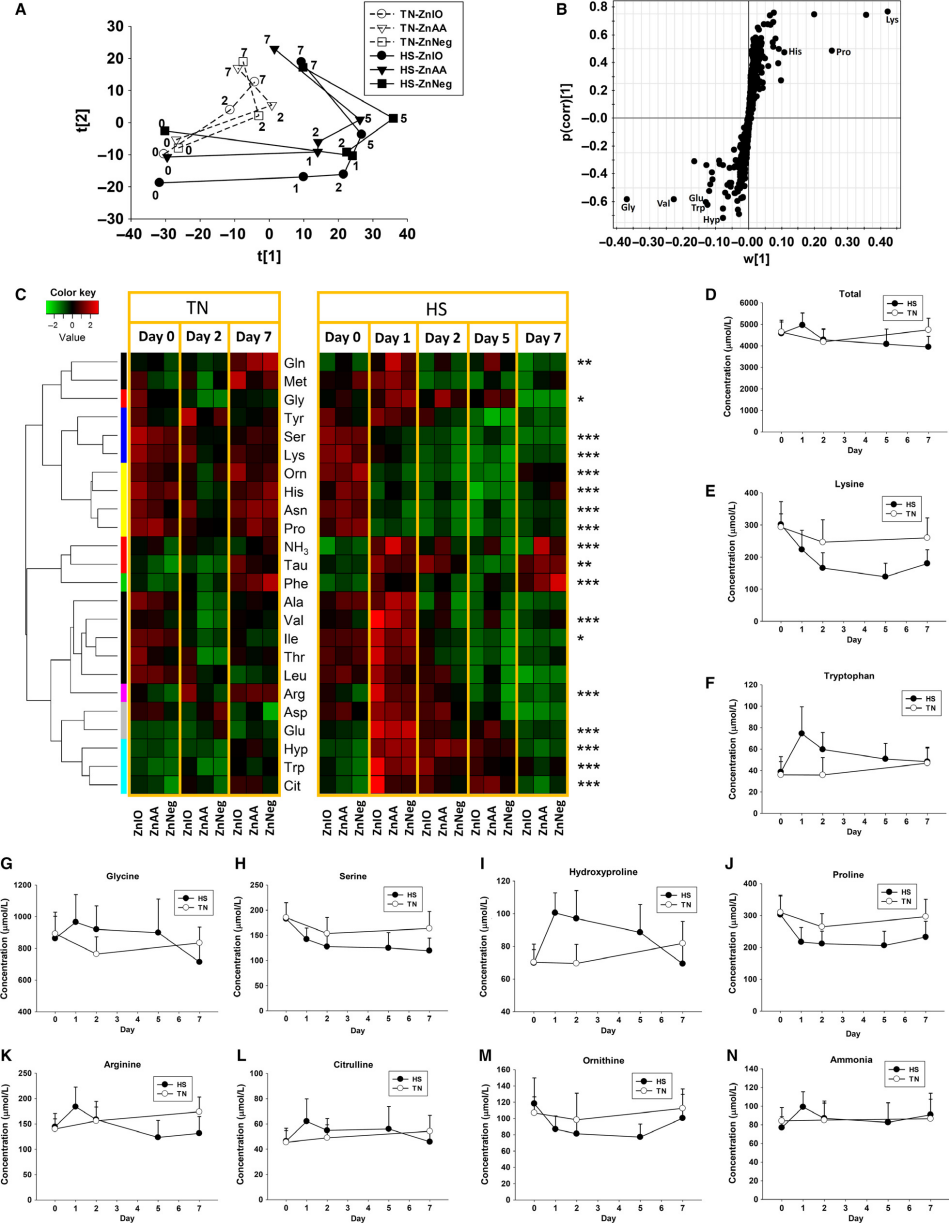
Metabolomic analysis of amino‐containing metabolites in serum during 1‐week HS and Zn supplementation. Amino‐containing metabolites were derivatized by DC prior to LC‐MS analysis. (A) The scores plot from PCA analysis on serum metabolome. The *t*[1] and *t*[2] values of each data point are the average scores of eight samples in principal components 1 and 2 of the model, respectively. These eight samples were harvested from the same treatment group on specified dates (marked in the plot) between day 0 and 7. (B) The S‐loadings plot on the ions contributing to the separation of HS and TN samples in an OPLS‐DA model. Major contributing metabolites are labeled. (C) The heat map on serum amino‐containing metabolites during 1‐week HS and Zn supplementation. The metabolites were grouped by HCA. Concentrations of each metabolite at different time points and in different sample groups were compared by its *Z* scores and are presented according to inlaid color keys. Significant differences between day 0 and day 1 in HS groups are determined by the Student's *t*‐test and labeled as *P <* *0.05, **P <* *0.01, and ***P <* *0.001. (D) The concentrations of total AAs in HS and TN groups. (E–N) The concentrations of lysine, tryptophan glycine, serine, hydroxyproline, proline, arginine, citrulline, ornithine, and ammonia in serum, respectively.

Metabolomic analysis was conducted to define the metabolite profile of serum samples collected at multiple time points during 1 week of diurnal HS challenge. Considering the observed changes in BUN and serum creatinine are associated with nitrogen metabolism (Fig. 2D–E), LC‐MS analysis and MDA of DC‐derivatized serum samples were performed to examine the influences of HS and Zn supplementation on the amino‐containing metabolites in serum. In the scores plot of an unsupervised PCA model, the principal component 1 was mainly defined by the differences between day 0 and day 1 HS samples, suggesting that HS induced dramatic changes in serum metabolome, especially on day 1 of HS (Fig. 3A). Day‐to‐day differences also existed among TN samples, but much less prominent than HS samples (Fig. 3A). Moreover, the influence of Zn supplementation was not apparent in the model since serum samples were not grouped based on the diets (Fig. 3A). The serum metabolites affected by HS exposure were identified in an S‐plot from a supervised OPLS‐DA model on HS and TN samples, showing major contributors to the separation of HS from TN samples are free AAs (FAAs) (Fig. 3B). The concentrations of major serum FAAs, including both proteinogenic and nonproteinogenic AAs, together with ammonia, were quantified and then examined by HCA. The distribution patterns of individual AAs in the heat map (Fig. 3C) and their concentrations (Fig. 3D–M and Table S3) resulted in the following conclusions on the effects of HS and Zn supplementation: (1) Majority of examined FAAs were affected by HS. In comparison, the FAA profile of TN samples was much more stable despite some day‐to‐day variances; (2) The most dramatic changes induced by HS occurred on day 1. Afterward, some of those changes were attenuated. This pattern of changes was consistent to both acute responses and the acclimation to HS, as shown by RT (Fig. 1C); (3) The level of total FAAs tended to increase on day 1 of HS (*P *=* *0.08) and then decreased afterward (Fig. 3D), but this pattern of change was not followed by individual FAAs. Among essential AAs, lysine and histidine were persistently decreased by HS (Fig. 3C and E), while tryptophan, valine, and isoleucine were transiently increased by HS (Fig. 3C and F). Among AAs that are directly associated by a specific metabolic reaction, glycine was increased (Fig. 3G), while serine, the precursor metabolite of glycine, was decreased by HS (Fig. 3H). Similarly, hydroxyproline was increased (Fig. 3I), while proline, its precursor, was decreased by HS (Fig. 3J). Among the metabolites associated with urea cycle and nitrogen balance, serum ammonia, arginine, citrulline, glutamate, and glutamine were increased while ornithine was decreased by HS (Fig. 3C and K–N).

**Figure 4:**
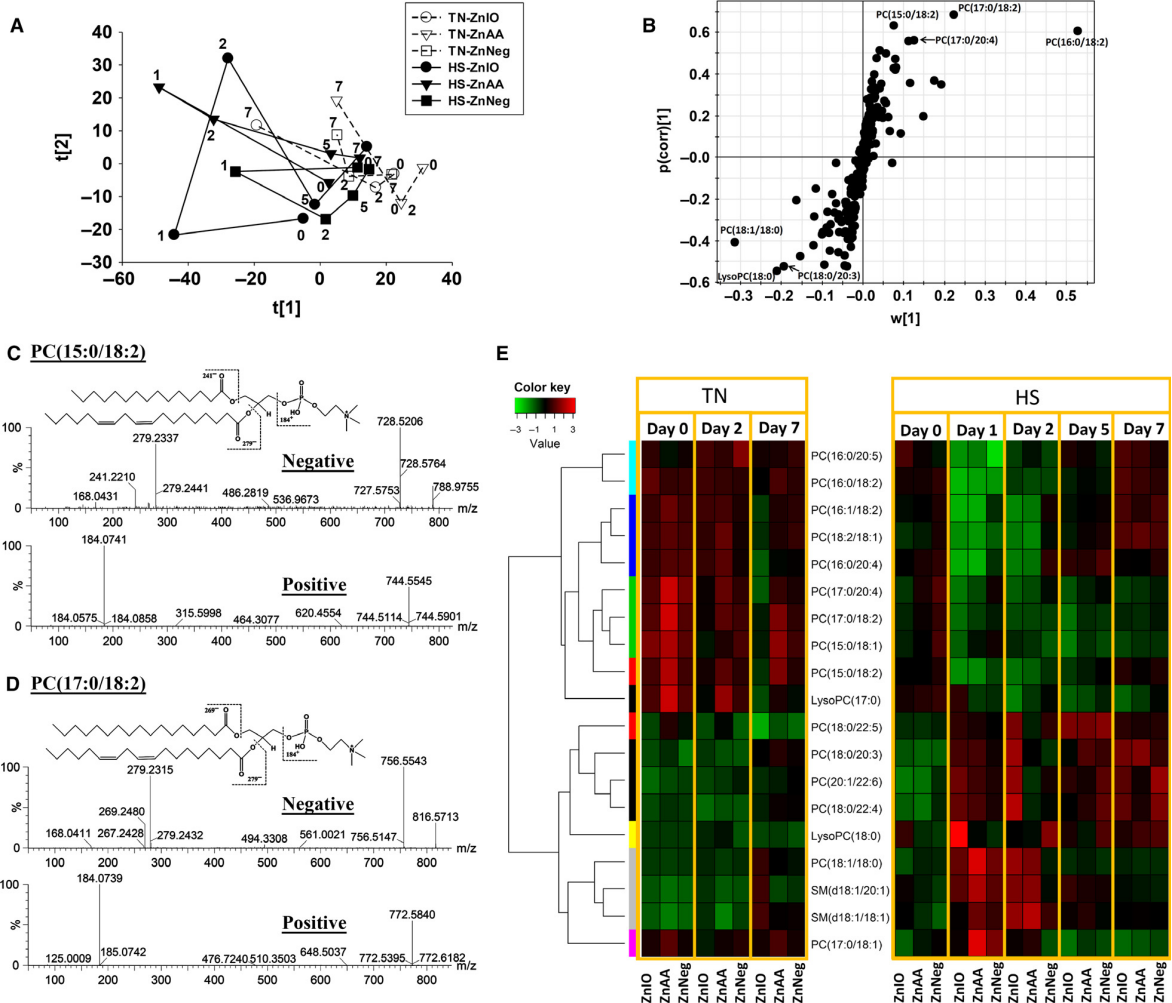
Metabolomic analysis of serum lipid profiles during 1‐week HS and Zn supplementation. (A) The scores plot from PCA analysis on serum metabolome. The *t*[1] and *t*[2] values of each data point are the average scores of eight samples in principal components 1 and 2 of the model, respectively. These eight samples were harvested from the same treatment group on specified dates (marked in the plot) between day 0 and 7. (B) The S‐loadings plot on the ions contributing to the separation of HS and TN samples in an OPLS‐DA model. Major contributing PCs are labeled. (C) Structure and MSMS spectrum of PC (15:0/18:2). (D) Structure and MSMS spectrum of PC (17:0/18:2). (E) The heat map on lipid species during 1‐week HS and Zn supplementation. Lipid species were grouped by HCA. Relative abundances of each lipid species at different time points and in different sample groups were compared by its *Z* scores and are presented according to inlaid color keys.

Besides amino‐containing metabolites, serum lipidome was also examined by LC‐MS analysis and MDA modeling. Based on the distribution pattern of examined samples in an unsupervised PCA model, HS induced dramatic changes in serum lipidome, especially on day 1 of exposure (Fig. 4A). Interestingly, Zn supplementation appeared to affect the metabolic responses to HS since HS‐induced changes occurred in ZnIO pigs did not resemble that changes in ZnAA and ZnNeg pigs (Fig. 4A). The lipids responsive to HS treatment were identified in the S‐plot of an OPLS‐DA model on HS and TN samples (Fig. 4B), and their identities as phospholipids, including phosphatidylcholines (PC), lysophosphatidylcholines (LysoPC), and sphingomyelins (SM), were defined by MSMS fragmentation. For example, the structures of PC (15:0/18:2) and PC (17:0/18:2) were determined by detecting the phosphocholine fragment in positive‐mode MSMS fragmentograms and the fatty acid fragments in negative‐mode MSMS fragmentograms (Fig. 4C–D). Based on their distribution patterns in the heat map generated by HCA, the serum lipid markers can be roughly classified into four groups, which are the phospholipids that were transiently decreased, persistently decreased, transiently increased, or persistently increased during 1 week of HS exposure (Fig. 4E). A prominent feature of those persistently decreased PCs is the presence of odd‐chain fatty acids (pentadecanoic acid and heptadecanoic acid) in their structures, while those persistently increased markers contain either very long‐chain fatty acid (carbon number ≥ 22) or stearic acid (Fig. 4E). Furthermore, two SM species were transiently increased in the first 2 days of HS, while multiple PC species containing palmitic acid or linoleic acid were transiently decreased by HS (Fig. 4E).

### Hepatic, cecal, fecal, and urinary metabolomes after 1‐week HS challenge

**Figure 5:**
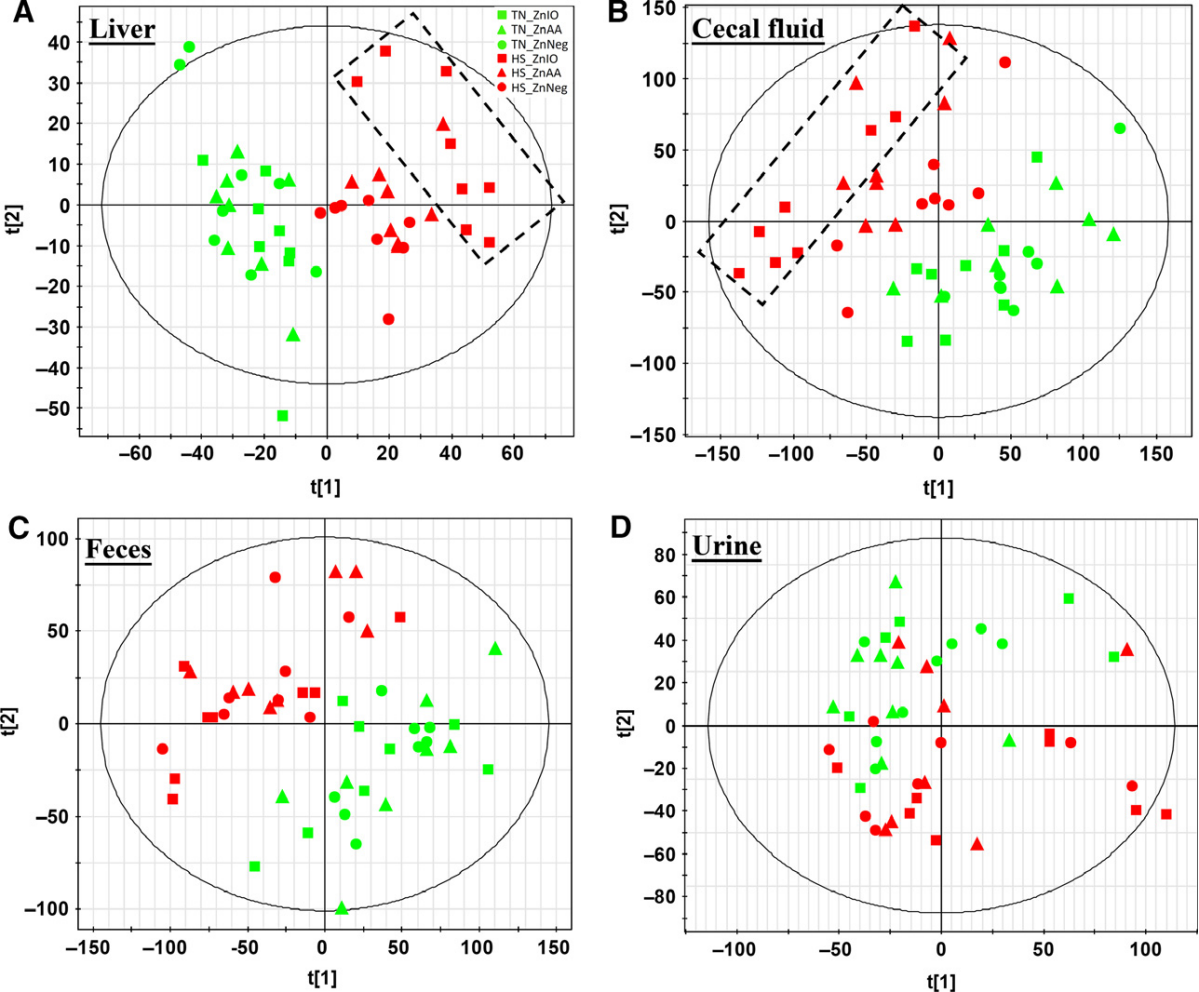
LC‐MS‐based metabolomic analysis of hepatic extract, cecal fluid, feces extract, and urine samples from pigs under HS and Zn supplementation. (A) Scores plot of a PCA model on hepatic extracts. The distribution of HS‐ZnIO samples in the model is illustrated by a rectangle. (B) Scores plot of a PCA model on cecal fluid samples. The distribution of HS‐ZnIO samples in the model is illustrated by a rectangle. (C) Scores plot of a PCA model on feces extract samples. (D) Scores plot of a PCA model on urine samples.

Compared with serum samples, the liver, cecal fluid, feces, and urine samples were only collected at the end of 1‐week HS. Therefore, metabolomic analyses of these samples revealed accumulative effects of HS exposure and Zn supplementation. Major classes of metabolites in these samples, such as bile acids, short‐chain fatty acids (SCFAs), lipids, and AAs, were analyzed by respective LC‐MS methods (detailed in Materials and Methods). Their LC‐MS data were pooled and then analyzed by PCA. Clear separations of HS and TN samples were observed in the PCA models on hepatic, cecal, and fecal metabolomes but not in the model on urine metabolome (Fig. 5A–D). Another noticeable feature is that HS‐ZnIO samples were more separated from TN samples than HS‐ZnAA and HS‐ZnNeg samples in PCA models of hepatic and cecal metabolomes (Fig. 5A–B). Markers contributing the separation of HS and TN samples were further characterized by elemental composition analysis, database, MSMS fragmentation, and confirmation with authentic standards if available, and then summarized based on their structures and biochemical functions (Table 1).

**Table 1:** Effects on HS and Zn supplementation on hepatic, cecal, fecal, and urinary metabolomes^a^

	Markers of HS	Markers of Zn supplementation
AAs & associated metabolites	*Increased by HS:* Creatinine^U^, Lys^L, F^, Cit^F^, Orn^F^ *Decreased by HS:* Ala^L^, Tyr^L^, Val^L^, His^L^, Asp^L^, Glu^L^, Met^L^, Ser^L^, Tau^L^, Pro^L^, Orn^L^, Ile^L^, Hyp^L^, kynurenic acid^U^	Lys^L^ (ZnIO > ZnAA, ZnIO > ZnNeg), Lys^F^ (ZnIO‐HS > ZnAA‐HS > ZnNeg‐HS)^b^
Phospholipids	*Increased by HS:* PE(16:0/18:2)^L^, PC(16:0/16:0)^L^, PC(16:0/18:2)^L^, PC(18:2/18:0)^L^, PC(18:0/20:3)^L^, PC(18:0/20:2)^L^, PC(18:0/22:6)^L^ *Decreased by HS:* PC(15:0/18:2)^L^, PC(15:0/18:1)^L^, PC(17:0/18:2)^L^, PC(17:0/18:1)^L^, PE(18:1/18:0)^L^, PE(18:1/20:4)^L^, PC(18:1/20:4)^L^, PC(18:0/22:3)^L^, LysoPE(16:0)^F^, LysoPC(16:0)^F^, LysoPC(18:2)^F^	PC(18:0/22:3)^L^ (ZnIO < ZnNeg), PC(16:0/18:2)^L^ (ZnIO‐HS > ZnNeg‐HS)^b^
Microbial metabolites	*Increased by HS:* Suberic acid^F^, sebacic acid^F^, hyodeoxycholic acid^C^, deoxycholic acid^C^, inosine^C^, acetic acid^C^, stercobilin^C, F^ *Decreased by HS:* Oleic acid^F^, linoleic acid^C, F^, oleamide^C^, xanthine^C^, 2,8‐dihydroxyquinoline^F^	Acetic acid^C^ (ZnIO > ZnAA, ZnIO > ZnNeg), Propionic acid^C^ (ZnIO > ZnNeg), Butyric acid^C^ (ZnIO > ZnAA, ZnIO > ZnNeg)

a Enlisted metabolites are the ones that were significantly affected by HS or Zn supplementation and also had their structures identified by either authentic standards or MSMS fragmentograms. The distribution of these markers in liver, cecum, feces, or urine was indicated by the superscripts “L”, “C”, “F”, “U”, respectively.

b *P *<* *0.05 from the PROC GLM procedure of SAS on the data of HS group.

#### AAs and associated metabolites (summarized in Table 1)

**Figure 6:**
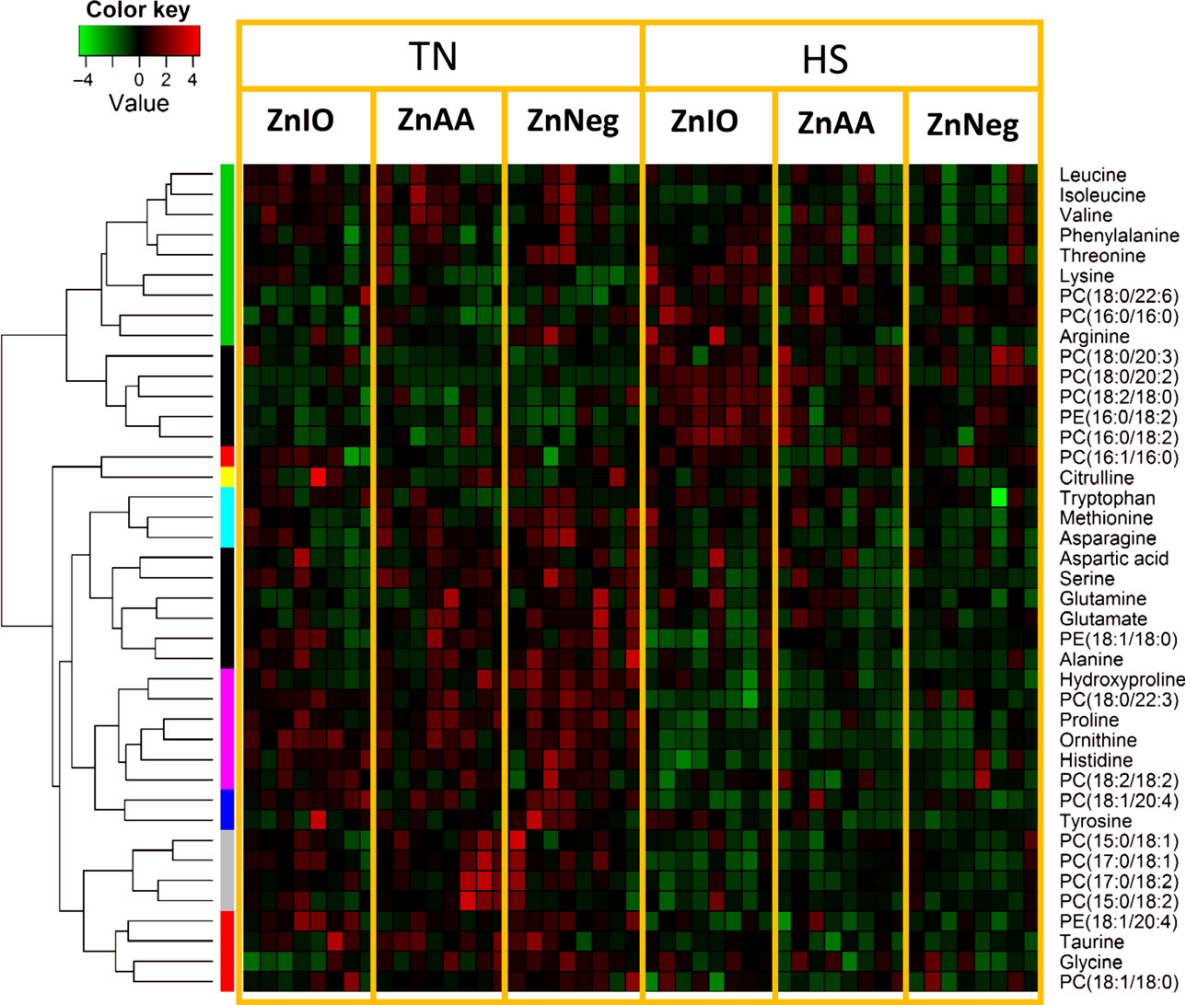
Metabolite markers of HS and Zn supplementation from LC‐MS‐based metabolomic analysis of hepatic extracts. The concentrations of FAAs and relative abundances of phospholipids in the liver are converted to the *Z* scores and presented in the heat map according to inlaid color keys. All enlisted metabolites have been confirmed by authentic standards or MSMS fragmentation.

The levels of many FAAs in the liver were decreased by HS, leading to the decrease of total FAAs in the liver (Fig. 6 and Table S4). In fact, this phenomenon is consistent with the decrease of total FAAs in serum on day 7 of HS (Fig. 3D). Opposite to this change in total FAAs, hepatic lysine levels were increased by HS (Fig. 6 and Table S4). However, the increase in lysine mainly occurred in ZnIO diet under HS. Interestingly, the higher level of lysine was also observed in ZnIO‐HS fecal samples (Fig. S1). Furthermore, HS also increased citrulline and ornithine in feces and creatinine in urine, but decreased kynurenic acid in urine (Figs. S1 and S2).

#### Fatty acids, phospholipids, and associated metabolites (summarized in Table 1)

**Figure 7:**
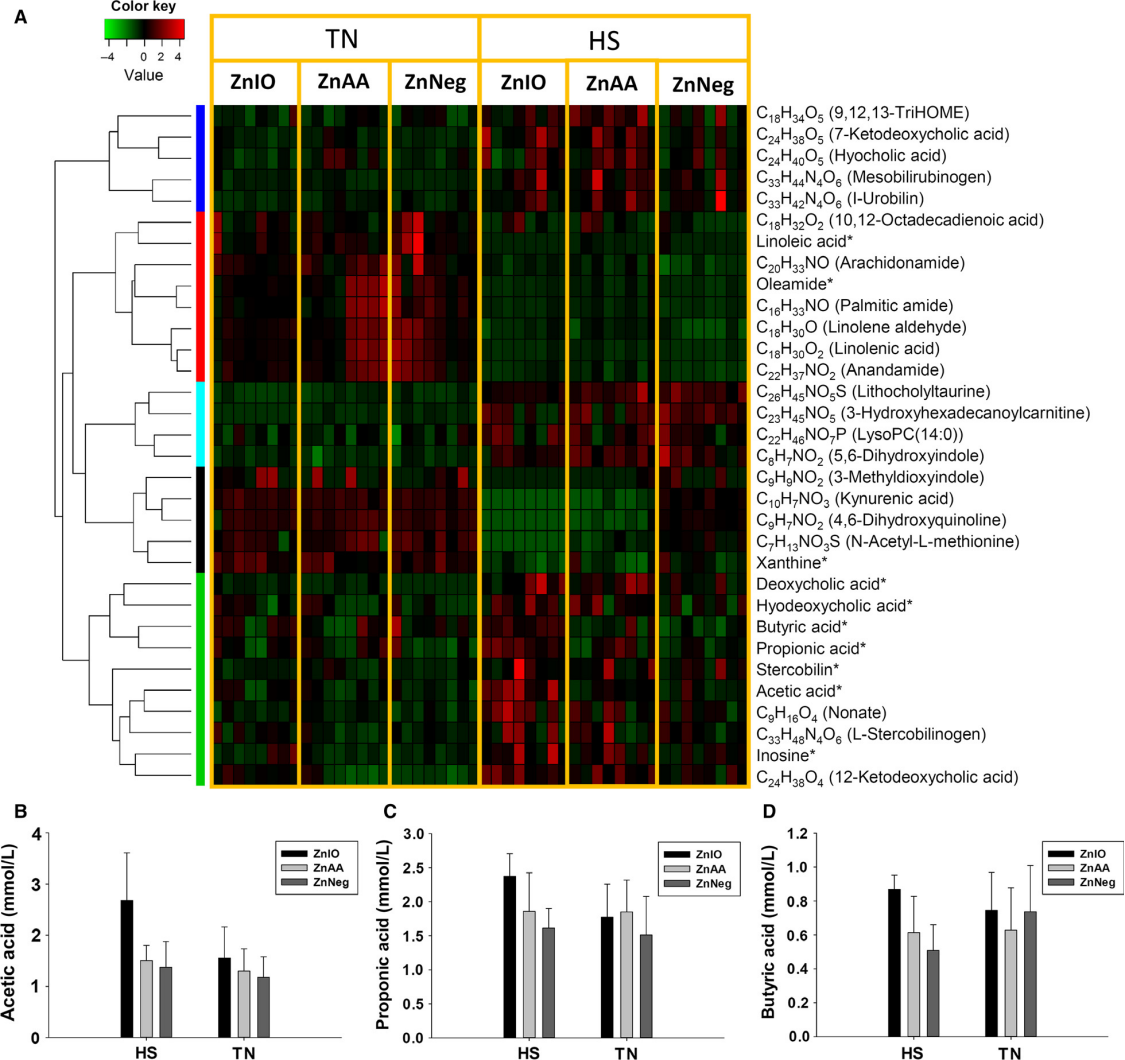
Metabolite markers of HS and Zn supplementation from LC‐MS‐based metabolomic analysis of cecal fluid. The markers labeled with * were confirmed with authentic standards. Putative identities of other markers were based on database search. (A) The heat map of cecal metabolite markers. The metabolites are grouped by HCA. Relative abundances of each metabolite across sample groups are converted to the *Z* scores and presented in the heat map according to inlaid color keys. (B) The concentration of acetic acid in cecal fluid. (C) The concentration of propionic acid in cecal fluid. (D) The concentration of butyric acid in cecal fluid.

Multiple PCs and phosphatidylethanolamines (PEs) in the liver were affected by HS (Fig. 6). Similar to the changes in serum lipidome (Fig. 4E), the PCs decreased by HS contain odd‐chain fatty acids (pentadecanoic acid and heptadecanoic acid), while the PCs increased by HS contain palmitic acid and stearic acid (Fig. 6). Moreover, in cecal and fecal samples, HS decreased the levels of oleic acid and linoleic acid, as well as fatty acid amide such as oleamide, but increased the levels of suberic acid and sebacic acid, which are medium‐chain dicarboxylic acids (Figs. 7A and S1).

#### Microbial metabolites (summarized in Table 1)

Stercobilin, a bacterial degradation product of bilirubin, was increased by HS in both cecal and fecal samples (Figs. 7A and S1). The levels of cecal hyodeoxycholic acid and deoxycholic acid, two major secondary bile acids in pigs, were also increased by HS (Fig. 7A). Interestingly, inosine, a purine nucleoside, was increased in cecal fluid by HS, accompanying with the decrease of xanthine, a degradation product of inosine (Fig. 7A). As major metabolites of microflora, SCFAs in cecal fluids were quantified. The higher concentrations of SCFAs, especially acetic acid and propionic acid, in ZnIO‐HS group suggested that ZnIO might promote SCFAs production under HS condition (Fig. 7B–D).

## Discussion

Metabolites are both the building blocks of animal growth and the regulators of animal health. Hence, metabolism is expected to play a central role in determining the influences of HS exposure and Zn supplementation on animal's growth performance and health status. Through metabolomic analysis in this study, diverse metabolites affected by HS and Zn were identified and characterized. The changes in these metabolites were treatment (HS vs. TN), diet (ZnIO vs. ZnAA vs. ZnNeg), or time (transient or persistent) specific. Discussions on the significance of these changes are based on the biochemistry of these metabolites, the physiology of HS and Zn supplementation, and metabolic functions of gut microflora.

### Effects of HS on swine metabolome

**Figure 8:**
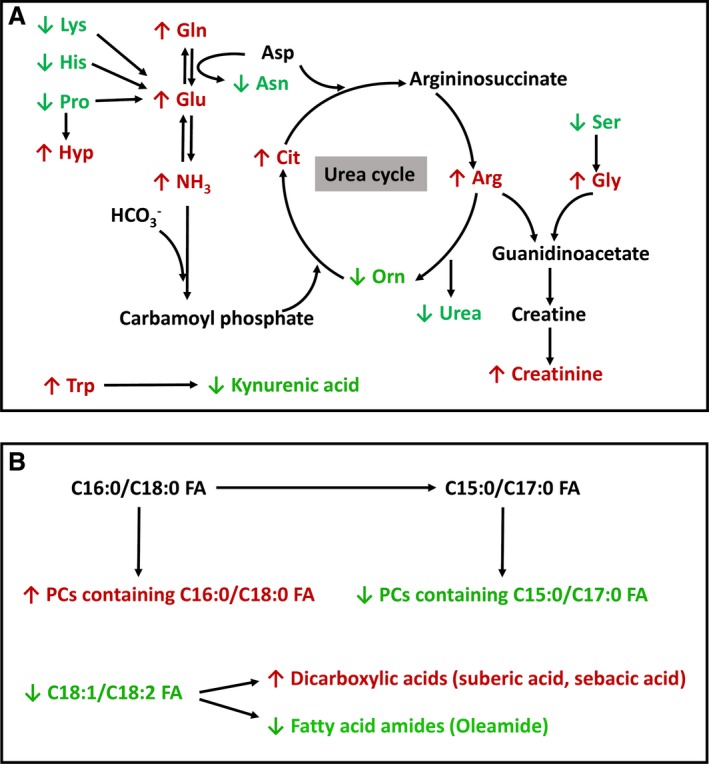
Summary of major HS‐induced metabolic changes. (A) HS‐induced changes in the metabolites related to nitrogen and amino acid metabolism. (B) HS‐induced changes in fatty acids and phospholipids.

Almost all biological processes in living entities, that is, humans, animals, plants, and microbes, are conducted within a narrow range of temperature. High ambient temperature affects energy and nutrient metabolism through its influences on thermogenesis and thermoregulation as well as the functions of biomolecules, such as enzymatic activity, protein folding, and lipid membrane integrity. In this study, the disruptive effects of HS on metabolic system were reflected indirectly by the changes in physiological parameters (RT and RR) and the decrease in growth performance (Fig. 1), and also directly by the metabolic changes detected by blood chemistry and metabolomic analysis (Figs. 2, 3, 4, 5, 6, 7). The results from metabolomic analysis of diverse samples, including serum, liver, cecal fluid, feces, and urine, not only reconfirmed the comprehensive impacts of HS on metabolic systems through multivariate modeling but also revealed previously unreported changes in AA, fatty acid, phospholipid, and microbial metabolism through identification and quantitation of metabolite markers (Figs. 3, 4, 5, 6, 7 and Table 1). Among these metabolites, FAAs, even though a minor fraction of total AA pool in the body, are useful indicators of nutritional and metabolic status since their levels are commonly controlled by dynamic equilibrium among different metabolic pathways (Abumrad and Miller 1983). Several interesting features were identified among many changes in FAAs and their associated metabolites: (1) Even though feed intake was reduced by HS, the total FAA level in serum actually tended to increase on day 1 of HS. Protein degradation, as a prominent consequence of HS, is likely contribute to this phenomenon. This conclusion is supported by the increase of creatinine in serum and urine after HS (Fig. 2E and Table 1). In addition, the increase of 4‐hydroxyproline, a major component of collagen, could be due to increased activity of collagenase under HS (Seltzer et al. 1989), while the increase of branched‐chain AAs (BCAAs: valine and isoleucine) could originate from hyperthermia‐induced muscle degradation as BCAAs account for one‐third of muscle proteins (Shimomura et al. 2004; Thomas and Crowhurst 2013); (2) Multiple metabolites associated with urea cycle and nitrogen metabolism were greatly affected by HS, especially on day 1 of HS. Urea (BUN) and ornithine (serum and liver), as the products of urea cycle, were decreased by HS. In contrast, the nitrogen donors and carriers, including glutamate (serum), ammonia (serum), citrulline (serum), and arginine (serum), were increased by HS (Fig. 8A). This pattern of changes suggests that HS can significantly disrupt urea cycle on day 1, potentially through negative regulation on arginase (Fig. 8A). It has been shown that HS‐induced vasodilation is correlated with the increase of nitric oxide biosynthesis (Kellogg et al. 2003), which in return could inhibit the activity of arginase (Daghigh et al. 1994). Whether this is a contributing mechanism on observed changes in urea cycle and nitrogen metabolism requires further studies; (3) Besides urea cycle, other changes in AA catabolism and biotransformation might also contribute to the changes in individual FAAs. For example, tryptophan in serum was greatly increased by HS (Fig. 3F), while kynurenic acid, a major catabolic metabolite of tryptophan, was decreased in urine (Table 1). Since kynurenine pathway connects these two metabolites, the downregulation of this pathway provides a plausible explanation for this observation. Furthermore, the decrease in proline and the increase of 4‐hydroxyproline could be contributed by the oxidation reaction between them, while the decrease in serine and the increase in glycine could also be associated with the one‐carbon metabolism between them. Further mechanistic studies are required to examine the validity of these hypothesis; (4) Lysine, as the first limiting AA in swine diet, was consistently decreased in serum during 1‐week HS (Fig. 3E), but its level in the liver and feces, especially in ZnIO‐HS pigs, was increased after HS (Fig. 6 and Table 1). This observation could be due to the changes in absorption and utilization of lysine or microflora‐mediated lysine biosynthesis in pigs (Torrallardona et al. 2003). Further studies are required to determine the underlying mechanism.

PCs, PEs, and SMs are major phospholipids of plasma membrane (Fadeel and Xue 2009). The observed changes in hepatic and serum phospholipids (Figs. 4 and 6, and Table 1) implicated that HS could disrupt plasma membrane through altering its composition, which is largely consistent to reported sensitivity of plasma membrane to HS (Torok et al. 2014). Examining the pattern of HS‐responsive phospholipid markers suggested that the transient changes in some of these markers resemble the time course of physiological response to HS, such as RT, which proceeded from acute response to acclimation (Fig. 1C). In contrast, the decrease of PCs containing pentadecanoic acid or heptadecanoic acid in serum was persistent during 7 days of HS (Fig. 4E), which was further confirmed by the phospholipid profile in the liver (Fig. 6 and Table 1). Considering microbial metabolism is a likely source of these two odd‐chain fatty acids (Jenkins et al. 2015), this pattern of change concurs with the lasting metabolic effects associated with the disruption of microflora (Weingarden et al. 2014). Furthermore, HS also increased multiple stearic acid‐containing PCs in the liver and serum. Since heptadecanoic acid could be formed by *α*‐oxidation of stearic acid (Foulon et al. 2005), it is possible that HS might suppress this biotransformation process, resulting in different distribution of fatty acids in phospholipids (Fig. 8B).

Besides observing altered fatty acid composition in phospholipids, examining cecal and fecal metabolomes also revealed the changes in fatty acid metabolism by observing the increases of medium‐chain dicarboxylic acids (suberic acid and sebacic acid) while the decreases of unsaturated fatty acids (oleic acid and linoleic acid) and their amides (oleamide) after HS (Table 1). Since medium‐chain dicarboxylic acids are the oxidation products of unsaturated fatty acids (Fujitani et al. 2009), the imbalance between these metabolites suggested that HS affects microflora‐mediated fatty acid metabolism (Fig. 8B).

The influences of HS on intestinal function and microbial metabolism were also reflected by other cecal and fecal metabolites (Table 1). For example, HS‐induced increases of secondary bile acids (hyodeoxycholic acid and deoxycholic acid) and bilirubin metabolites (stercobilin and stercobilinogen) could be due to reduced reabsorption in the gut or more active microbial metabolism. Moreover, the increase of inosine and the decrease of xanthine in cecal samples suggest purine degradation in microflora was negatively affected by HS.

### Effects of Zn supplementation on swine metabolome

Even though higher than the dietary requirement for growing pigs (50 ppm for pigs weighing 50–100 kg) (NRC 2012), Zn supplementation (120 ppm) in the present study did not significantly affect serum Zn level (Fig. 2F), growth performance (Fig. 1E–G), and blood chemistry (Fig. 2A–E). The lack of diet‐induced changes in serum Zn level could be due to the facts: (1) the Zn supplementation in this study adopted a more physiologically relevant dose and a shorter duration compared with other Zn supplementation experiments that increased serum Zn level (Schell and Kornegay 1996; Borah et al. 2014); and (2) there exists robust regulation of Zn homeostasis in the body (King et al. 2000). Previous studies have shown that serum and whole‐body Zn levels in humans and animals are relatively stable even under a wide range of dietary Zn intake, since higher Zn intake is commonly translated into reduced absorption efficiency and higher fecal excretion (Weigand and Kirchgessner 1978, 1980; Cragg et al. 2005). Therefore, significant change in serum Zn level may only occur under extremely low or high Zn intake when insufficient Zn input or Zn overload alters the exchangeable pool in Zn homeostasis (King 1990). These two scenarios did not occur in this study since 30 ppm Zn was detected in ZnNeg diet, while 120 ppm Zn in ZnIO and ZnAA diets is not expected to overwhelm the body.

Even though HS‐induced metabolic changes are dominant in defining the differences among sample groups in this study, Zn supplementation also led to subtle metabolic differences among pigs under HS challenge. This conclusion is based on the MDA models of examined metabolomes and the HS‐induced changes in individual metabolites. A prominent feature is that ZnIO diet appeared to elicit stronger metabolic responses to HS than ZnAA and ZnNeg diets. As shown in both unsupervised PCA models of hepatic and cecal metabolomes (Fig. 5A–B), HS‐ZnIO samples were further away from TN samples than HS‐ZnAA and HS‐ZnNeg samples. Moreover, the PCA model of serum lipids (Fig. 4A) also showed that the time‐dependent changes in HS‐ZnIO samples differed from the change in HS‐ZnAA and HS‐ZnNeg samples, especially in the first 2 days of HS. On individual metabolites, HS‐induced increase of serum tryptophan in ZnIO pigs was much greater than that in ZnAA and ZnNeg pigs (Table S3). Furthermore, under HS, ZnIO pigs had higher level of lysine in the liver and feces than ZnAA and ZnNeg pigs (Fig. 6 and Table 1). HS also increased SCFAs, especially acetic acid and propionic acid, in cecal fluids of ZnIO pigs, but not with ZnAA and ZnNeg pigs (Fig. 6B–D and Table 1). Since SCFAs in cecal fluid originate from microbial fermentation, this observation suggested that ZnSO_4_ in ZnIO diet might elevate these fermentation activities, especially the formation of acetic acid. The mechanism behind the selective effects of ZnIO diet requires further investigation. However, the function of inorganic Zn, including ZnO and ZnSO_4_, as modulation agents for the stability of microflora and the growth of pathogenic microbes have been shown in previous studies (Katouli et al. 1999; Surjawidjaja et al. 2004).

### Values and challenges of metabolomics

Targeted analyses of specific metabolites have been performed in numerous studies on HS exposure and Zn supplementation (Baumgard and Rhoads 2013; Borah et al. 2014; Belhadj Slimen et al. 2015). Despite their merits in confirming expected metabolic changes or revealing general metabolic status of study subjects, targeted analyses are incapable of defining global profile of metabolic system or identifying unexpected metabolic events. Adopting untargeted metabolomics in this study addressed these limits of targeted analyses, resulting in the identification of AAs, lipids, and microbial metabolites responsive to HS challenge or Zn supplementation. The procedures of LC‐MS‐based untargeted metabolomics platform, which include sample preparation, LC‐MS analysis, MDA, marker identification, and characterization, have been extensively reviewed (Wang and Chen 2013; Gika et al. 2014). In this study, the values and challenges of individual metabolomic analysis procedures in discovering metabolite markers are evident and can be summarized as follows. (1) In sample preparation, besides adopting respective extraction methods for each type of samples, chemical derivatization, using DC and HQ, had greatly extended the coverage of LC‐MS analysis and facilitated the quantitation of metabolite markers in this study. Considering chemical derivatization is a requisite step in GC‐MS‐based metabolomics, the application of chemical derivatization in LC‐MS‐based metabolomics remains limited and can be greatly expanded (Xu et al. 2011); (2) PCA and HCA were adopted to process complex LC‐MS data in this study. Besides revealing the treatment‐ and diet‐associated grouping of examined samples, PCA modeling was also able to show time‐dependent events, including acute response and acclimation to HS, based on sample distribution in the score plots (Figs. 3A and 4A). Subsequently, the metabolite markers identified by PCA were processed by HCA, which produced the heat maps revealing both the correlations among multiple as well as time‐dependent changes (Figs. 3C and 4E). This combination of PCA and HCA facilitated data visualization and marker identification and is an efficient approach for examining complex metabolomics datasets; (3) Despite progresses in constructing metabolomic databases and bioinformatics tools, marker identification remains a bottleneck in many metabolomics efforts (Wishart 2011). In this study, accurate mass‐based database search suggested the candidate structures with the same elemental composition. However, unambiguous structural identification of these metabolite markers was not achieved until performing comparative analysis of authentic standards or MSMS fragmentation, which often corrected the structure proposed by initial analysis. Since many recent published metabolomics works heavily relied on database search‐based structural identification, the experience in this study highlights the need to be cautious when processing the results from database search.

In conclusion, comprehensive metabolomic analysis of diverse biological samples in this study revealed dramatic HS‐induced metabolic effects and subtle Zn supplementation‐associated metabolic changes in growing pigs. Changes in identified metabolite markers of HS and Zn supplementation indicate that diurnal HS disrupted nitrogen homeostasis, AA, lipid, and microbial metabolism, while Zn supplementation, especially ZnIO, affected microbial metabolism under HS. All these observations warrant further investigations on the causes of identified metabolic events as well as their roles in physiological responses to HS and Zn supplementation.

## Conflict of Interest

None declared.

## Supporting information




**Table S1.** Ingredients and formulation of three experimental diets.
**Table S2.** Measured concentrations of minerals in premixes and diets.
**Table S3.** Serum FAAs concentration.
**Table S4.** Liver FAAs concentration.
**Figure S1.** Metabolite markers of HS and Zn supplementation from LC‐MS‐based metabolomic analysis of fecal extracts. The markers labeled with * were confirmed with authentic standards. Putative identities of other markers were based on database search. The metabolites are grouped by HCA. Relative abundances of each metabolite across sample groups are converted to the *Z* scores and presented in the heat map according to inlaid color keys.
**Figure S2.** Metabolite markers of HS and Zn supplementation from LC‐MS‐based metabolomic analysis of urine samples. The markers labeled with * were confirmed with authentic standards. Putative identities of other markers were based on database search. The metabolites are grouped by HCA. Relative abundances of each metabolite across sample groups are converted to the *Z* scores and presented in the heat map according to inlaid color keys.
