# Supplementary material for: Metabolomics revealed diurnal heat stress and zinc supplementation‐induced changes in amino acid, lipid, and microbial metabolism
Source: Physiol Rep. 2016 Jan 12;4(1):e12676. doi: 10.14814/phy2.12676 (PMC4760408; doi:10.14814/phy2.12676)
Supplement: Supplementary file 1 — Table S1. Ingredients and formulation of three experimental diets. Table S2. Measured concentrations of minerals in premixes and diets. Table S3. Serum FAAs concentration. Table S4. Liver FAAs concentration. Figure S1. Metabolite markers of HS and Zn supplementation from LC‐MS‐based metabolomic analysis of fecal extracts. The markers labeled with * were confirmed with authentic standards. Putative identities of other markers were based on database search. The metabolites are grouped by HCA. Relative abundances of each metabolite across sample groups are converted to the Z scores and presented in the heat map according to inlaid color keys. Figure S2. Metabolite markers of HS and Zn supplementation from LC‐MS‐based metabolomic analysis of urine samples. The markers labeled with * were confirmed with authentic standards. Putative identities of other markers were based on database search. The metabolites are grouped by HCA. Relative abundances of each metabolite across sample groups are converted to the Z scores and presented in the heat map according to inlaid color keys. [file PHY2-4-e12676-s001.docx]

**SUPPORTING INFORMATION**

**Table S1.**Ingredientsandformulation of three experimental diets

|  |  | Diet |  |
| --- | --- | --- | --- |
| Ingredients(%) | ZnIO | ZnAA | ZnNeg |
| Corn | 88.94 | 88.94 | 88.94 |
| Soybeanmeal | 8.41 | 8.41 | 8.41 |
| L-lysineHCl | 0.30 | 0.30 | 0.30 |
| Monocalciumphosphate | 0.71 | 0.71 | 0.71 |
| Limestone | 0.75 | 0.75 | 0.75 |
| Salt | 0.40 | 0.40 | 0.40 |
| Vitamin premix | 0.25 | 0.25 | 0.25 |
| Trace mineral premix* | 0.24 | 0.24 | 0.24 |
| Total | 100.00 | 100.00 | 100.00 |
| **Trace mineral premix* |  |  |  |
| *Non-zinc trace mineral premix*^†^ | *0.12* | *0.12* | *0.12* |
| *Zinc sulfate, monohydrate* | *0.12* | *0.06* | *-* |
| *Zinc-AA (AvailaZn^TM^)* | *-* | *0.06* | *-* |
| *Carrier (rice hull)* | *-* | *-* | *0.12* |

^†^To provide the followings per kg of diet: Fe, 100 mg;Mn, 10 mg; Cu, 10 mg;Iodine, 0.20 mg; Se, 0.30 mg.

**Table S2.** Measured concentrations of minerals in premixes and diets

|  | Trace mineral premix | | | | | | Diet | | | | | |
| --- | --- | --- | --- | --- | --- | --- | --- | --- | --- | --- | --- | --- |
| Mineral | ZnIO | | ZnAA | | ZnNeg | | ZnIO | | ZnAA | | ZnNeg | |
| Calcium | 16.95 | % | 13.57 | % | 21.81 | % | 0.66 | % | 0.57 | % | 0.60 | % |
| Copper | 0.78 | % | 0.56 | % | 1.14 | % | 11.58 | ppm | 12.86 | ppm | 14.93 | ppm |
| Iron | 5.54 | % | 6.28 | % | 9.59 | % | 207.10 | ppm | 221.80 | ppm | 256.40 | ppm |
| Magnesium | 0.46 | % | 0.40 | % | 0.61 | % | 0.11 | % | 0.11 | % | 0.11 | % |
| Manganese | 0.81 | % | 0.70 | % | 1.11 | % | 21.92 | ppm | 19.29 | ppm | 18.17 | ppm |
| Phosphorus | 0.03 | % | 0.05 | % | < 0.02 | % | 0.42 | % | 0.45 | % | 0.48 | % |
| Potassium | 0.22 | % | 0.16 | % | 318.00 | ppm | 0.46 | % | 0.45 | % | 0.44 | % |
| Sodium | 893.70 | ppm | 632.40 | ppm | 467.10 | ppm | 0.17 | % | 0.16 | % | 0.15 | % |
| Zinc | 8.79 | % | 7.20 | % | 0.10 | % | 153.90 | ppm | 142.90 | ppm | 29.90 | ppm |

**Table S3.**Serum FAAs concentration

|  | Groups | | | | | | Treatments | | Diets | | | *P*^†^ | | |
| --- | --- | --- | --- | --- | --- | --- | --- | --- | --- | --- | --- | --- | --- | --- |
| FAAs (µM) | TN-ZnIO | TN-ZnAA | TN-ZnNeg | HS-ZnIO | HS-ZnAA | HS-ZnNeg | TN | HS | ZnIO | ZnAA | ZnNeg | T x D | Diet | Trt |
| Alanine | 505.53 | 494.13 | 484.73 | 448.41 | 504.87 | 447.87 | 494.72^a^ | 466.33^b^ | 476.13 | 499.50 | 465.91 | 0.142 | 0.153 | 0.049 |
| Glycine | 865.34 | 828.97 | 807.14 | 823.98 | 904.51 | 876.29 | 833.64 | 867.93 | 844.53 | 866.33 | 841.36 | 0.073 | 0.637 | 0.142 |
| Valine | 196.59 | 189.82 | 182.82 | 197.28 | 208.49 | 184.34 | 189.67 | 196.45 | 196.94^ab^ | 198.94^a^ | 183.57^b^ | 0.266 | 0.021 | 0.168 |
| Phenylalanine | 85.47 | 81.30 | 85.47 | 85.47 | 92.59 | 89.29 | 84.03^a^ | 89.29^b^ | 85.47 | 86.96 | 86.96 | 0.101 | 0.830 | 0.022 |
| Tyrosine | 59.37 | 48.31 | 50.20 | 48.44 | 40.28 | 39.81 | 52.52^a^ | 42.75^b^ | 53.76^a^ | 44.20^b^ | 44.85^b^ | 0.901 | 0.001 | <0.0001 |
| Tryptophan | 44.37 | 35.17 | 35.14 | 64.34 | 52.82 | 55.38 | 38.10^a^ | 57.41^b^ | 53.89^a^ | 43.54^b^ | 44.69^b^ | 0.893 | 0.0004 | <0.0001 |
| Arginine | 168.28 | 152.15 | 141.43 | 158.5 | 150.78 | 142.52 | 153.96 | 150.60 | 163.39^a^ | 151.47^ab^ | 141.98^b^ | 0.651 | 0.003 | 0.505 |
| Histidine | 85.17 | 84.26 | 86.69 | 63.58 | 72.89 | 70.75 | 85.38^a^ | 69.07^b^ | 74.38 | 78.57 | 78.72 | 0.055 | 0.067 | <0.0001 |
| Lysine | 302.45 | 266.87 | 256.49 | 182.66 | 193.12 | 158.64 | 275.27^a^ | 178.14^b^ | 242.56^a^ | 230.00^ab^ | 207.57^b^ | 0.095 | 0.004 | <0.0001 |
| Aspartic acid | 7.93 | 8.70 | 7.53 | 8.07 | 8.44 | 7.25 | 8.05 | 7.91 | 8.00 | 8.57 | 7.39 | 0.886 | 0.053 | 0.728 |
| Asparagine | 96.14 | 90.78 | 87.66 | 65.67 | 72.46 | 62.29 | 91.49^a^ | 66.74^b^ | 80.18 | 81.37 | 74.43 | 0.168 | 0.073 | <0.0001 |
| Glutamate | 130.99 | 139.83 | 139.17 | 169.83 | 176.94 | 154.42 | 136.61^a^ | 166.80^b^ | 149.16 | 157.29 | 146.60 | 0.273 | 0.370 | <0.0001 |
| Glutamine | 562.32 | 579.86 | 564.63 | 536.71 | 599.80 | 553.06 | 568.91 | 562.88 | 549.44^a^ | 589.79^b^ | 558.84^ab^ | 0.360 | 0.037 | 0.649 |
| Methionine | 28.36^a^ | 23.49^b^ | 25.94^ab^ | 22.30^b^ | 25.11^ab^ | 23.76^b^ | 25.89^a^ | 23.71^b^ | 25.23 | 24.30 | 24.84 | 0.002 | 0.674 | 0.012 |
| Serine | 175.83 | 168.45 | 164.53 | 128.56 | 133.15 | 122.06 | 169.58^a^ | 127.88^b^ | 151.27 | 150.29 | 142.51 | 0.526 | 0.191 | <0.0001 |
| Threonine | 71.81 | 59.87 | 61.69 | 62.20 | 58.59 | 56.88 | 64.25 | 59.19 | 66.83 | 59.22 | 59.24 | 0.508 | 0.031 | 0.056 |
| Taurine | 82.32 | 74.34 | 72.62 | 90.38 | 88.10 | 84.34 | 76.36^a^ | 87.59^b^ | 86.30 | 81.07 | 78.37 | 0.725 | 0.111 | 0.0004 |
| Proline | 283.35 | 299.23 | 282.05 | 204.36 | 228.10 | 205.37 | 288.10^a^ | 212.34^b^ | 240.64^a^ | 261.26^b^ | 240.66^a^ | 0.688 | 0.023 | <0.0001 |
| Citrulline | 51.82 | 49.49 | 43.49 | 58.82 | 52.69 | 51.81 | 48.20^a^ | 54.40^b^ | 55.27^a^ | 51.08^ab^ | 47.56^b^ | 0.434 | 0.002 | 0.0005 |
| Ornithine | 111.40 | 99.78 | 106.79 | 85.34 | 86.19 | 80.01 | 105.89^a^ | 83.80^b^ | 97.50 | 92.74 | 92.43 | 0.201 | 0.374 | <0.0001 |
| Isoleucine | 88.62 | 78.52 | 80.67 | 73.13 | 70.22 | 62.97 | 82.49^a^ | 68.63^b^ | 80.50 | 74.25 | 71.27 | 0.548 | 0.140 | 0.0004 |
| Leucine | 222.43 | 220.75 | 217.45 | 209.39 | 214.95 | 195.88 | 220.21^a^ | 206.74^b^ | 215.91 | 217.85 | 206.67 | 0.478 | 0.186 | 0.012 |
| Hydroxyproline | 73.06 | 74.55 | 68.09 | 87.19 | 86.15 | 86.90 | 73.06^a^ | 86.75^b^ | 79.81 | 80.14 | 76.92 | 0.356 | 0.439 | <0.0001 |
| Total | 4339.64 | 4198.64 | 4101.42 | 3934.00 | 4186.94 | 3881.05 | 4213.24^a^ | 4000.66^b^ | 4136.82 | 4192.79 | 3991.24 | 0.149 | 0.118 | 0.010 |

*The FAA concentrations are the means of serum samples belonging to the same groups, treatments, or diets.

^†^Statistical significance is calculated by thePROC MIXED procedure of SAS.

^a,b,c^Withingroups, treatments or diets, the values of individual AAs with different letter labels are statistically different(*P* <0.05).

**Table S4.** Liver FAAs concentration

|  | Groups | | | | | | Treatments | | Diets | | | *P*^†^ | | |
| --- | --- | --- | --- | --- | --- | --- | --- | --- | --- | --- | --- | --- | --- | --- |
| FAAs | TN-ZnIO | TN-ZnAA | TN-ZnNeg | HS-ZnIO | HS-ZnAA | HS-ZnNeg | TN | HS | ZnIO | ZnAA | ZnNeg | T x D | Diet | Trt |
| Alanine | 2405.47 | 2430.86 | 2754.52 | 2166.35 | 2079.54 | 2092.89 | 2525.51^a^ | 2112.65^b^ | 2282.67 | 2248.46 | 2400.90 | 0.318 | 0.486 | 0.0004 |
| Glycine | 2722.50^a^ | 3098.50^ab^ | 3171.00^b^ | 3034.60^ab^ | 2910.30^ab^ | 2876.90^ab^ | 2997.33 | 2940.60 | 2878.55 | 3004.40 | 3023.95 | 0.010 | 0.306 | 0.496 |
| Valine | 330.40 | 369.80 | 358.00 | 350.80 | 361.10 | 330.90 | 362.93^a^ | 337.37^b^ | 355.90 | 350.10 | 344.45 | 0.576 | 0.713 | 0.029 |
| Phenylalanine | 154.00 | 153.50 | 162.20 | 160.70 | 157.80 | 158.90 | 156.57 | 159.13 | 157.35 | 155.65 | 160.55 | 0.789 | 0.806 | 0.679 |
| Tyrosine | 86.10 | 81.62 | 111.46 | 86.59 | 54.33 | 53.97 | 92.19^a^ | 63.32^b^ | 86.35 | 66.59 | 77.56 | 0.143 | 0.364 | 0.015 |
| Tryptophan | 48.30 | 42.10 | 45.40 | 49.60 | 46.30 | 40.00 | 45.27 | 45.30 | 48.95 | 44.20 | 42.70 | 0.351 | 0.164 | 0.990 |
| Arginine | 45.67 | 48.50 | 58.85 | 61.81 | 45.23 | 45.40 | 50.70 | 50.25 | 53.13 | 46.84 | 51.69 | 0.0499 | 0.506 | 0.924 |
| Histidine | 663.30 | 639.90 | 677.00 | 521.30 | 572.10 | 593.10 | 660.07^a^ | 562.17^b^ | 592.30 | 606.00 | 635.05 | 0.268 | 0.195 | <0.0001 |
| Lysine | 278.70 | 240.30 | 236.10 | 324.50 | 281.80 | 259.80 | 251.70^a^ | 288.70^b^ | 301.60^a^ | 261.05^b^ | 247.95^b^ | 0.736 | 0.002 | 0.004 |
| Aspartic acid | 982.70 | 1175.09 | 1156.55 | 1041.69 | 1031.43 | 978.58 | 1101.25 | 1016.89 | 1011.81 | 1100.92 | 1063.80 | 0.389 | 0.624 | 0.268 |
| Asparagine | 410.30^ab^ | 457.20^ab^ | 497.80^a^ | 429.10^ab^ | 364.80^b^ | 373.80^b^ | 455.10^a^ | 389.23^b^ | 419.70 | 411.00 | 435.80 | 0.026 | 0.641 | 0.004 |
| Glutamate | 2971.10 | 3503.30 | 3802.20 | 2918.00 | 2926.30 | 2985.70 | 3425.53^a^ | 2943.33^b^ | 2944.55 | 3214.80 | 3393.95 | 0.225 | 0.138 | 0.011 |
| Glutamine | 2543.51 | 2965.79 | 3076.97 | 2934.52 | 2775.82 | 2547.84 | 2852.64 | 2748.20 | 2732.03 | 2869.23 | 2800.07 | 0.042 | 0.747 | 0.479 |
| Methionine | 83.20 | 89.50 | 101.90 | 88.00 | 80.90 | 77.60 | 91.53^a^ | 82.17^b^ | 85.60 | 85.20 | 89.75 | 0.032 | 0.641 | 0.037 |
| Serine | 974.30 | 1089.90 | 1135.00 | 883.20 | 861.10 | 831.20 | 1066.44^a^ | 858.50^b^ | 928.75 | 975.50 | 983.10 | 0.218 | 0.628 | 0.0001 |
| Threonine | 206.30 | 216.50 | 235.20 | 233.90 | 205.80 | 201.80 | 219.33 | 213.83 | 220.10 | 211.15 | 218.50 | 0.033 | 0.703 | 0.555 |
| Taurine | 1142.30 | 1217.80 | 1116.33 | 1025.67 | 889.54 | 949.94 | 1158.06^a^ | 953.46^b^ | 1082.47 | 1040.75 | 1029.78 | 0.188 | 0.660 | 0.0002 |
| Proline | 620..70 | 700.00 | 693.10 | 467.30 | 458.80 | 447.20 | 671.27^a^ | 457.77^b^ | 544.00 | 579.40 | 570.15 | 0.434 | 0.658 | <0.0001 |
| Citrulline | 17.90 | 15.50 | 15.00 | 13.60 | 10.80 | 11.80 | 16.13 | 12.07 | 15.75 | 13.15 | 13.40 | 0.960 | 0.574 | 0.073 |
| Ornithine | 482.80 | 435.28 | 440.41 | 291.55 | 280.00 | 283.13 | 452.37^a^ | 284.86^b^ | 375.18 | 349.11 | 353.12 | 0.856 | 0.498 | <0.0001 |
| Isoleucine | 268.50 | 278.20 | 264.20 | 218.90 | 220.10 | 222.80 | 270.30^a^ | 220.60^b^ | 243.70 | 249.15 | 243.50 | 0.766 | 0.854 | <0.0001 |
| Leucine | 503.80 | 484.10 | 479.40 | 494.30 | 475.00 | 464.20 | 489.10 | 477.83 | 499.05 | 479.55 | 471.80 | 0.983 | 0.320 | 0.456 |
| Hydroxyproline | 74.20^ac^ | 87.20^ab^ | 98.90^b^ | 73.50^ac^ | 67.00^c^ | 70.60^ac^ | 86.77^a^ | 70.37^b^ | 73.85^a^ | 77.10^ab^ | 84.75^b^ | 0.008 | 0.042 | <0.0001 |
| Total | 18174 | 19945 | 20873 | 18009 | 17235 | 16983 | 19664^a^ | 17409^b^ | 18092 | 18590 | 18928 | 0.025 | 0.461 | 0.0002 |

*Unit is nmol/g liver.

^†^Statistical significance is calculated bythe PROC MIXED procedure of SAS.

^a,b,c^Withingroups, treatments or diets, the values of individual AAs with different letter labels are statistically different(*P* <0.05).


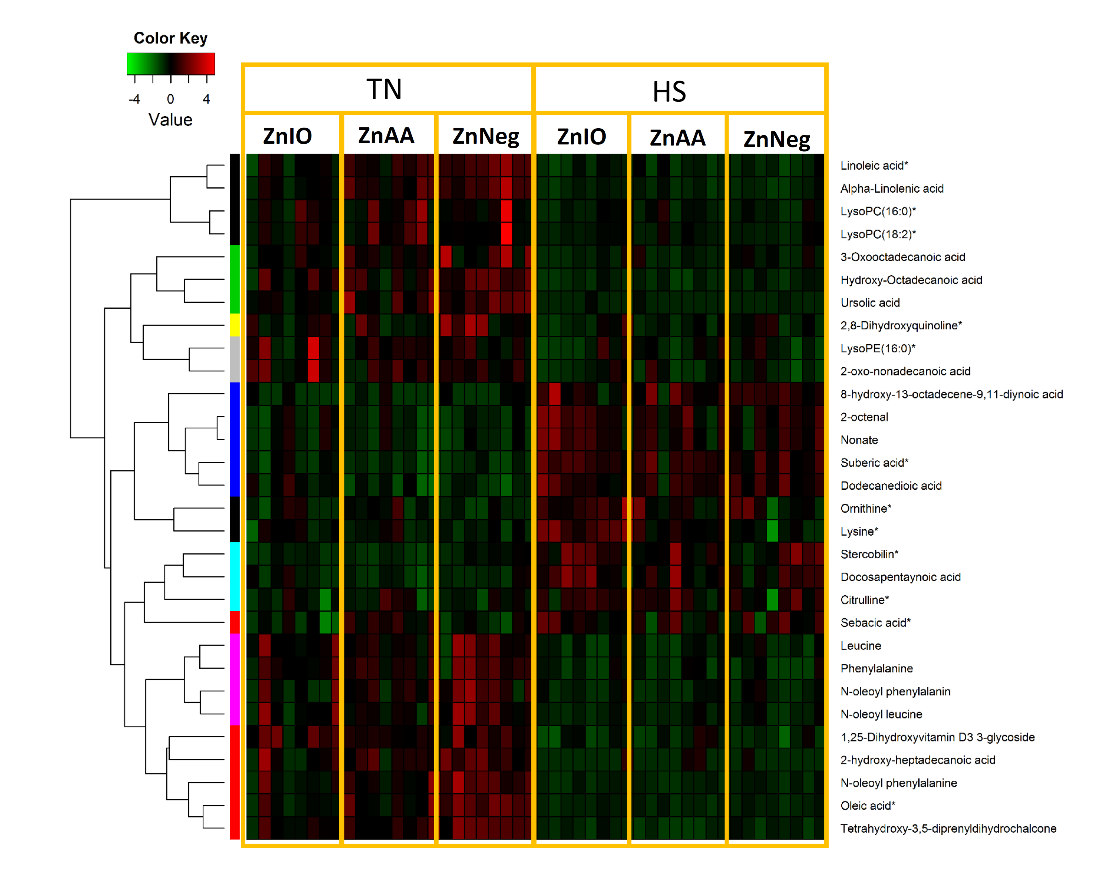


**Fig. S1.**Metabolite markersof HS and Zn supplementation from LC-MS-based metabolomic analysis of fecal extracts. The markers labeled with * were confirmed with authentic standards. Putative identities of other markers were based on database search. The metabolites are grouped by HCA. Relative abundances of each metaboliteacross sample groups are converted to the Z scores and presented in the heat map according to inlaid color keys.


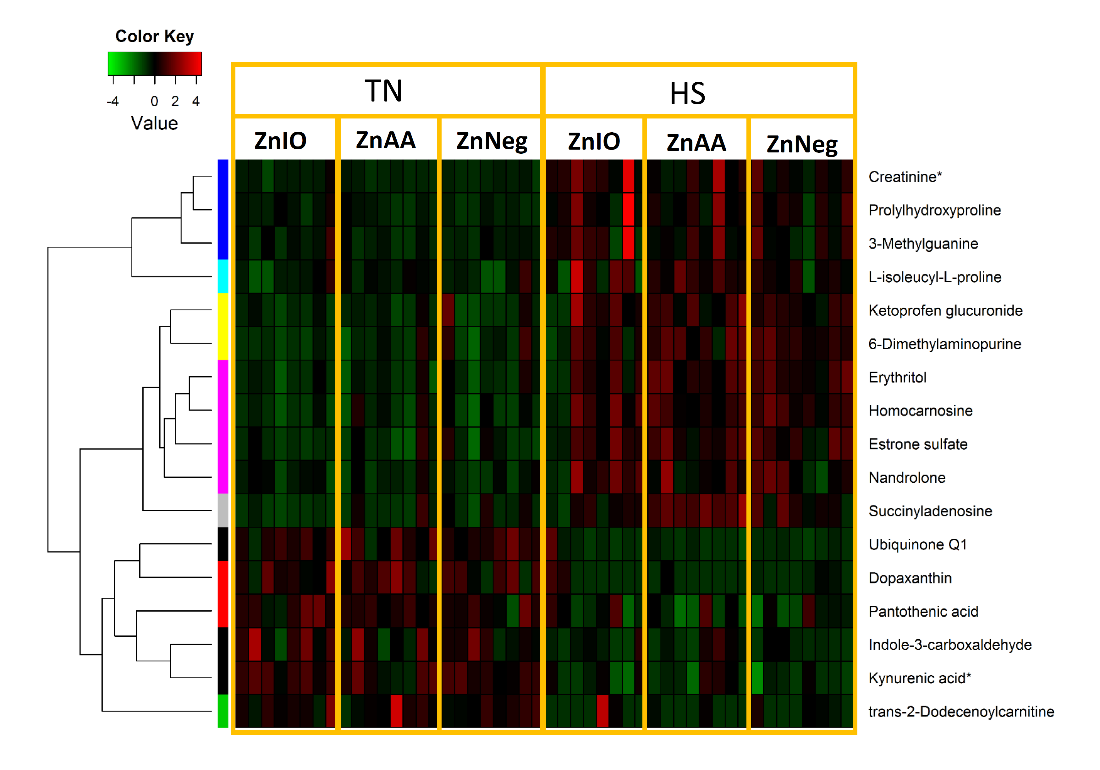


**Fig. S2.**Metabolite markersof HS and Zn supplementation from LC-MS-based metabolomic analysis of urine samples. The markers labeled with * were confirmed with authentic standards. Putative identities of other markers were based on database search. The metabolites are grouped by HCA. Relative abundances of each metaboliteacross sample groups are converted to the Z scores and presented in the heat map according to inlaid color keys.
